# In vitro expansion of fetal liver hematopoietic stem cells

**DOI:** 10.1038/s41598-021-91272-6

**Published:** 2021-06-04

**Authors:** Rashmi Bhardwaj, Lalit Kumar, Deepika Chhabra, N. K. Mehra, Atul sharma, Sujata Mohanty, Vinod Kochupillai

**Affiliations:** 1 Institute Rotary Cancer Hospital (IRCH), All India Institute of Medical Sciences (AIIMS), New Delhi, India; 2 Sri Sri Institute For Advanced Research (SSIAR), Ved Vignan Maha Vidhya Peeth (VVMVP), F003 Soudhamini Apartment, 21st Kanakpura Road, Art of Living International Center, Udaipura, Bengaluru, 560082 India

**Keywords:** Stem cells, Medical research

## Abstract

Fetal liver hematopoietic stem and progenitor cells (HSPCs) have been considered appropriate for the management of aplastic anemia owing to their proliferative potential. Bone marrow recovery was possible in some cases; the engraftment potential of these cells, however was unsatisfactory, possibly due to the availability of a smaller number of these cells from a single fetus. The present study explores how we can expand fetal liver hematopoietic stem cells under in vitro conditions. We isolated mononuclear cells from fetal liver and hematopoietic stem cells were identified and analyzed by cell surface marker CD34. CD34^+^ fetal liver HSPCs cells were separated by magnetic cell sorting positive selection method. HSPCs (CD34^+^) were cultured by using 5 cytokines, stem cell factor (SCF), granulocyte macrophages-colony stimulating factor (GM-CSF), interleukin-6 (IL-6), Fms-related tyrosine kinase 3 (FLT-3) and erythropoietin (EPO), in 4 different combinations along with supplements, in serum-free culture media for 21 days. Cell viability continued to be greater than 90% throughout 21 days of culture. The cells expanded best in a combination of media, supplements and 5 cytokines, namely SCF, FLT-3, IL6, EPO and GM-CSF to yield a large number of total (CD34^+^ & CD34^-^) cells. Even though the total number of nucleated cells increased in culture significantly, levels of CD34 antigen expression declined steadily over this period.

## Introduction

Hematopoiesis appears in the fetal liver (FL) at approximately 5 weeks of gestation and remains the primary site of hematopoiesis until mid-gestation^1^. The HSPCs rapidly proliferate in the liver; undergo maturation and differentiation leading to erythropoiesis, myelopoiesis, B and T lymphopoiesis, and production of megakaryocytes^2^. Thereafter, the bone marrow replaces the fetal liver as the primary site of hematopoiesis^3^. HSPCs have been used to treat a number of nonmalignant conditions such as Aplastic Anemia (AA), Severe Combined Immunodeficiency Disease (SCID), Congenital Metabolic Disorders, and also malignant conditions such as Acute Myeloid Leukemia (AML), Acute Lymphoblastic Leukemia (ALL), Myelodysplastic Syndrome (MDS), Chronic Myeloid Leukemia (CML), and Multiple Myeloma (MM). Fetal liver cells have the advantage of better growth and differentiation because of their higher telomere content^4^. Also, the proportion of HSPCs (marked by surface antigen CD 34) in the fetal liver are greater than those found in cord blood and bone marrow; and are more or less equal to those found in mobilized peripheral blood^5^. Fetal liver hematopoietic stem and progenitor cells (FL-HSPCs) have been found to exhibit greater proliferative potential compared to the adult bone marrow and cord blood. Increased capacity in serial transfer, greater tritiated thymidine uptake index, evidenced this along with early appearance of the peak number of colonies in semi-solid assays and greater plating efficiency^6,7,8,9^. It was also found that these cells lack proper Human Leukocyte Antigen (HLA) expression^10^. With having all the above-mentioned properties, fetal liver infusion has been utilized for the management of aplastic anemia^11^. A limited quantity of fetal liver cells obtained from a single abortus however, creates problems in carrying out different experiments in the laboratory. A regular supply of the FL-HSPCs, probably can be obtained from expanded cells. Expanded fetal liver CD34^+^ HSPCs produced under ‘‘in vitro*’’* conditions may provide large homogenous population of cells from a single fetal liver source.

## Material and methods

### Ethical approval

The Human Ethics Committee (Institutional Review Board) of All India Institute of Medical Sciences, New Delhi, India approved the current study. The Patient Information sheet was shared and we obtained Informed consent from patients who underwent Medical Termination of Pregnancy (MTP). We confirm that we carried all methods as per the relevant guidelines and regulations.

### Preparation of cell suspension and cell viability

We obtained human fetuses with gestation periods ranging from 8 to 20 weeks with no known chromosomal abnormality following medical termination of pregnancy (MTP) with Prostaglandin E or Emcradyl injection from the Department of Obstetrics and Gynecology at AIIMS. Immediately after expulsion, we collected fetuses on ice and brought to the Laboratory. We dissected the fetuses under aseptic conditions to separate the liver. The liver was placed into Iscove’s Modified Dulbecco Medium (IMDM) containing 10% fetal bovine serum (FBS) and 10 IU/ml heparin. Further, the liver was mashed into small pieces and allowed to pass through sterile muslin cloth repeatedly to obtain single-cell suspension. 10 µl of the fetal liver cell suspension was mixed with 10 µl of Trypan blue dye and loaded onto a hemocytometer and the number of dye excluding cells (viable) were counted. Only those fetus samples which had 80% or more cell viability were taken up for further analysis.

### Isolation of fetal liver mononuclear cells (MNC)

Mononuclear cells from fetal liver nucleated cell suspension were separated using density gradient centrifugation for their subsequent use in culture studies. After 5 min of treatment with 1X Red Cell lysis buffer (Sigma) to remove mature erythrocytes and PBS washing, nucleated cells from fetal liver suspension were diluted with IMDM containing 10% FBS in the ratio of 1:3 (fetal liver suspension: IMDM with FBS) and 30 ml of this mix was gently layered on 10 ml of Histopaque-1077 (Sigma Aldrich) in 50 ml sterile centrifuge tubes. The mononuclear cells were isolated by centrifugation (30 min, 1500 rpm, at room temperature) in a swinging-bucket rotor without brake. The white cell ring appearing at the interface of the medium and consisting of mononuclear cells (MNCs) was carefully withdrawn with a fine pasture pipette. MNCs so obtained were washed thrice in phosphate buffer saline (PBS) to remove any traces of Histopaque and cells were counted on a hemocytometer before labeling with magnetic bead or fluorochrome-conjugated Antibodies.

### Isolation of fetal liver hematopoietic stem and progenitor cells (CD34^+^ Cells) by MACS

The CD34^+^ HSPCs fraction was isolated immunomagnetically using MS^+^ MiniMACS columns and the CD34 Direct Isolation Kit (Miltenyi Biotec, USA) as per manufatcurer’s instructions. Aftre removal from MS coloumns and washing with PBS, the MACS sorted HSPCs were analysed for surface antigen CD34 through flow cytometer (BD FACS Calibur) using CD34PE antibody (BD Biosciences). Statistics were elaborated in 10,000 events/sample by WinMDI software. Purified CD34 + cells from MACS sorting were used for cell culture study.

### Expansion of CD34^+^ FL-HSPCs under ‘in vitro’ conditions

We followed Stroma-free long-term culture technique to expand the FL- HSPCs. Freshly isolated 1X10^4^ FL CD 34^+^ cells were cultured in a flat bottomed 24 well plates in 1 ml of serum-deprived culture medium STEM PRO-34-SFM (Gibco, Thermo Fisher) supplemented with 100 µg/ml insulin (Sigma), 200 µg/ml transferrin (Sigma), 40 µg/ml low density lipoprotein (LDL) (Sigma), 2 mM Glutamine (Gibco), 10^-4^ M Mercaptoethanol (Sigma). Different cytokine combinations (Table 1) comprising SCF (100 ng/ml), FLT-3(100 ng/ml), IL-6(20 ng/ml), EPO (2U/ml) and GM-CSF (200U/ml), (Peprotech) were used to expand CD34^+^ HSPCs for a period of 21 days. Fetal liver CD34^+^ HSPCs cultures were grown at 37 °C in humidified 5% CO2 incubator. Wells were demi- depopulated once a week. Harvested cells were counted and suitable aliquots were assayed every seven days for the presence of specific surface antigen CD34 by flow cytometry (BD FACS Calibur).

**Table 1:** Combinations of Cytokines used for expansion of fetal liver hematopoietic stem and progenitor cells.

Cytokine used in Combinations	Combination 1 (M)	Combination 2 (M + S)	Combination 3 (M + S + M1)	Combination 4 (M + S + M2)	Combination 5 (M + S + M3)	Combination 6 (M + S + M4)
Media	Media	Media	Media	Media	Media	Media
Supplements		Supplements	Supplements	Supplements	Supplements	Supplements
SCF			100 ng/ml	100 ng/ml	100 ng/ml	100 ng/ml
FLT-3			100 ng/ml	100 ng/ml	100 ng/ml	100 ng/ml
IL-6				20 ng/ml	20 ng/ml	20 ng/ml
EPO					2U/ml	2U/ml
GM-CSF						200U/ml

M = Media, S = Supplements, M1 = SCF + Flt-3, M2 = SCF + Flt-3 + IL6, M3 = SCF + Flt-3 + IL6 + EPO, M4 = SCF + Flt-3 + IL6 + EPO + GM-CSF.

### Colony forming unit assay

Colony Forming Unit (CFU assay) was performed from the in vitro expanded CD34^+^ HSPCs. 1 × 10^4^ cells were taken from the expanded culture of Fetal liver CD34^+^ HSPCs at an interval of 7 days and mixed with methylcellulose semisolid medium (MethoCult H4434). Colony-forming unit-granulocyte macrophage (CFU-GM), Colony-forming unit-granulocyte magakaryocyte macrophage (CFU-GEMM), and Blast-forming unit- erythroid (BFU-E) were scored with an inverted microscope at day 14 of the culture.

### Statistical analysis

All data about fetuses and culture characteristics were entered and analyzed using Microsoft excel and SPSS software. Statistical analysis was performed to determine the significance between two culture populations with minimum of three replicates. Multivariate analysis ANOVA was done between groups to determine statistical significance (**P ≤ 0.01 and ***P ≤ 0.001).

## Results

### Cell number and viability range of human fetal liver

The age of the fetuses varied from 8–20 weeks (Median = 12 weeks). The median number of total fetal cells was 1.77 × 10^9^ (Range; 0.02–5.2 × 10^9^) with mean viability of 93.81 ± 5%. Total viable mononuclear cells obtained from fetal liver nucleated (Non Hepatocytic) cell suspension using density gradient centrifugation were 2.27 ± 1.8 1X 10^8^ (Range; 0.01–6.78 X1 0^8^) (Table 2).

**Table 2:** Fetal liver characteristics at the time of collection.

Characteristics	Number
Total Fetuses collected	70
Sex Male: Female	39 (55.7%): 31 (44.2%)
**Age (Gestation Period)**	
Mean ± SD	13 ± 2.91 weeks
Median	12 weeks
Range	8–20 weeks
**Time between expulsion and processing of Fetus**	
Mean	1.00 ± 0.76 h
Range	0.25–3.5 h
**Non hepatocytic cell viability of liver cells**	
Mean	93.81 ± 5.0%
Range	85–100%
**Total viable Nucleated (Non Hepatocytic) Cells**	
Mean ± SD	1.91 ± 1.31 × 10^9^
Median	1.77 × 10^9^ 0.02-
Range	5.2 × 10^9^
**Total viable fetal liver mononuclear cells**	
Mean ± SD	2.27 ± 1.81 × 10^8^
Median	2.041 × 10^8^
Range	0.01–6.78 × 10^8^

### High CD34 expression on human fetal liver mononuclear cells

**Figure 1:**
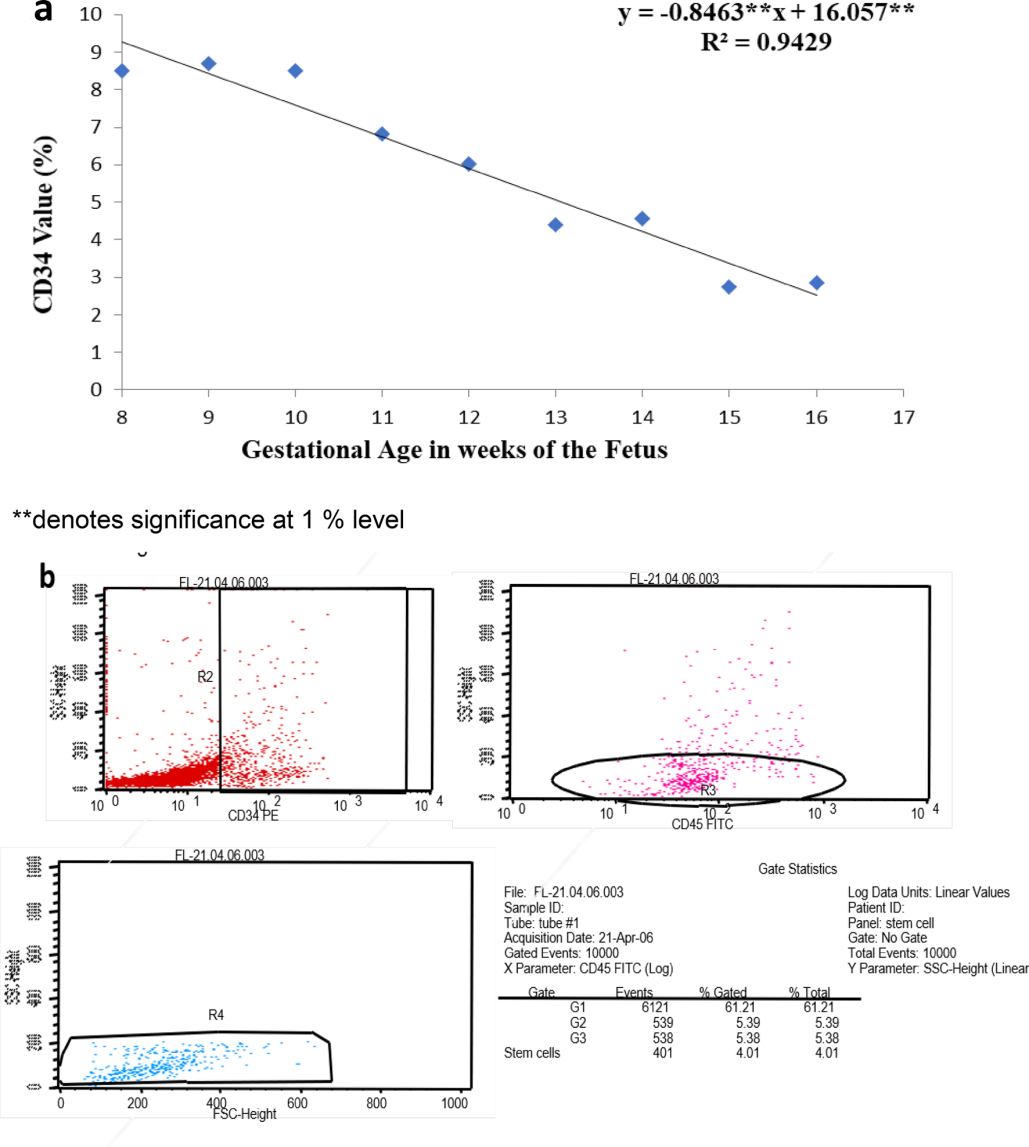
(**a**) Percentage of CD34^+^ cells vs. Gestation age of the fetus. CD34^+^ population among fetal liver mononuclear cells ranged from 1.2% -12.8% in 8–16 weeks (n = 3 for each week) gestational age of fetuses (**a**) Statistical significance was analyzed via ANOVA and is depicted with asterisks (*). double (**) asterisks indicates *P* ≤ 0.01. Representative primary immunophenotypic data of CD34 + cells (also tested and positive for CD45) from fetal liver mononuclear cells showing 5.39% of total evaluated cells expressing CD34 antigen (**b**).

Fetal liver MNCs through flowcytometry showed that the total CD34^+^ HSPCs population ranged from 1.2% to 12.8% with a median value of 5.5%. Supplementary Table 1 displays the percentage of CD34^+^ cells among FLMNCs according to gestational age. The peak values of CD34^+^ HSPCs were observed during 8–16 weeks of gestational age (Fig. 1a). From 8 to 10th week, CD34^+^ percentage remained high (8.50 ± 2.4% to 8.70 ± 1.8%); subsequently it started declining. We performed multivariate linear regression analysis on our results which shows that increase in gestational age of 1 week changes CD34 percentage by 0.8463 units. The overall regression model was significant and depicted 1% level of significance with a coefficient of determination (R^2^) value at 0.9429.

### CD34^+^ HSPCs purification

The total no. of CD34^+^ HSPCs separated by MACS positive selection method ranged from 5.20 × 10^5^–4.34 × 10^6^ to (median 2.79 × 10^6^) with a median recovery of 62.3% (range 31.8–75.3%). The median purity of CD34^+^ HSPCs isolated from MiniMACS was 95% (Range 89–98%). In all samples, the percentage cell viability of CD34^+^ isolated HSPCs were greater than 95% as measured by Trypan blue dye exclusion methods. CD34^+^ cell fraction is nonadherent while CD34^-^ cells adhere to the flask in culture media and this fraction of cells may contain fetal liver stromal cells^12^.

### 137 fold expansion of CD34^+^ fetal liver HSPCs

**Figure 2:**
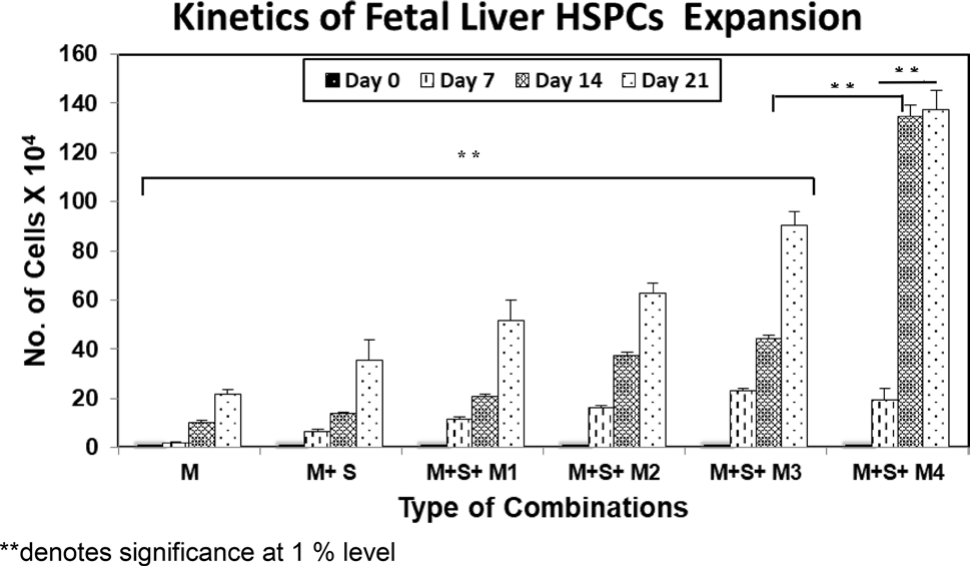
Kinetics of fetal liver CD34 + HSPCs expansion Estimated cell counts at 7-days intervals from day 0–24. Expansion of total cells expressed as fold increase over initial cell input in all the tested combinations. Results are expressed as mean ± SEM of 5 experiments. Statistical significance was analyzed via ANOVA and is depicted with asterisks (*). double (**) asterisks indicate *P* ≤ 0.01. The increase in number of cells in different combinations (M vs M + S + M3 and M3 vs M4) as well as at different days (day 7 vs Da 21) in M + S = M4 shows significant increase with *P* ≤ 0.01.

Estimated cell counts at 7 days intervals are shown in Fig. 2. Cell viability throughout 21 days of culture was greater than 90%. Expansion of total cells was expressed as an increase over initial cell input. Total nucleated cells expanded up to 137.24 ± 8.1 fold (M + S + M4) by day 21 (*p* < 0.01). A multivariate approach was adapted by performing ANOVA to assess the difference in cell expansion between different culture conditions. Difference between average number of cells in M and M + S + M4 was highly significant (-64.3555) as p value (0.000) < 0.01 with 95% confidence level of interval. Highest average number of cells was found in combination M + S + M4 followed by M + S + M3 with mean difference significant at the 0.05 level. Analysis was also performed based on different timepoints and it was found that even at earlier timepoints of Day 7 and Day 14, difference between expanded average numbers was highly significant with *p*-value (0.000) < 0.01 with 95% confidence level of interval.

### Declining CD34^+^ expression on expanded FL-HSPCs

**Figure 3:**
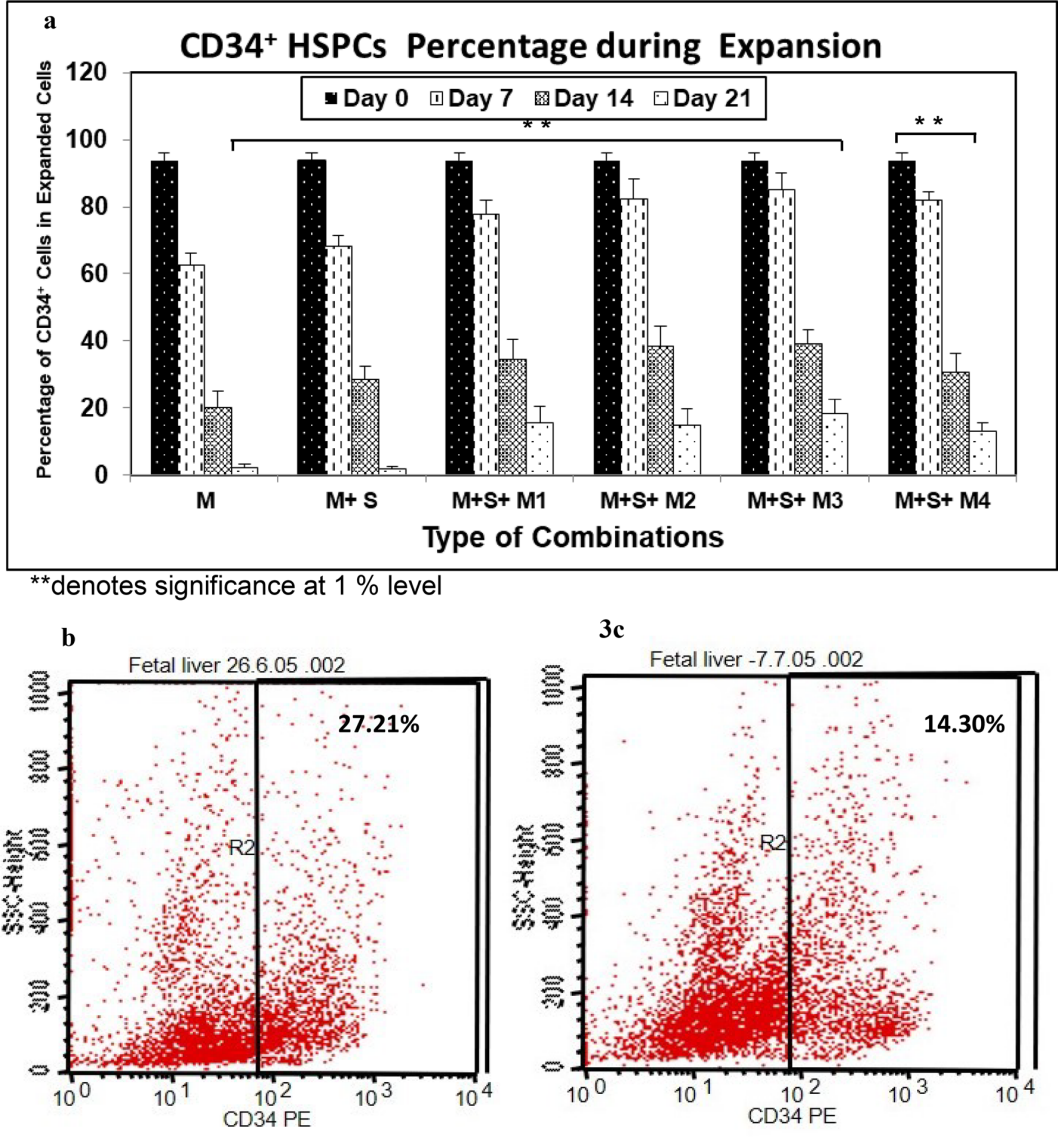
Percentage fetal liver CD34^+^ HSPCs cells during 21 days of expansion: CD34 expression of in vitro expanded cells from day 0–21 done through flowcytometry. Mean cell percentage is indicated (n = 5) with SEM (**a**). Statistical significance among different combinations as well as days was analyzed via ANOVA and is depicted with asterisks (*). double (**) asterisks indicate *P* ≤ 0.01. (**b**) Representative image of immunophenotyping of CD34 antigen from in vitro expanded cells using M + S + M4 media and cytokines combination at Day 14 (**b**) and Day 21(**c**) done through flowcytometry.

Expanded cells observed from day 0 to day 21, the percentage of CD34^+^ cells declined quickly after day seven and continued to decline steadily over the twenty-one days of culture. FACS analysis of CD34 expression of five representative experiments at different time points is shown in Fig. 3a. The Percentage of CD34^+^ among the total nucleated cell count at day 21 was 15.42 ± 4.9 in combination M + S + M1, 14.68 ± 5.1% in M + S + M2, 18.24 ± 4.2% in M + S + M3, and 13.08 ± 2.3% in M + S + M4, however, in media (M) alone it was 1.7 ± 2.0%. ANOVA analysis was adapted to assess the significance levels of difference in decline of CD34 expression between different culture conditions. The decline in CD34 + HSPCs percentage was found to be highly significant among different combination groups with *p*-value (0.000) < 0.01 with 95% confidence level of interval. Absolute number of total cells and CD34% cells at day 21 are given in Supplementary Table 2.

### Colony forming unit assay from in vitro expanded FL-HSPCs

**Figure 4:**
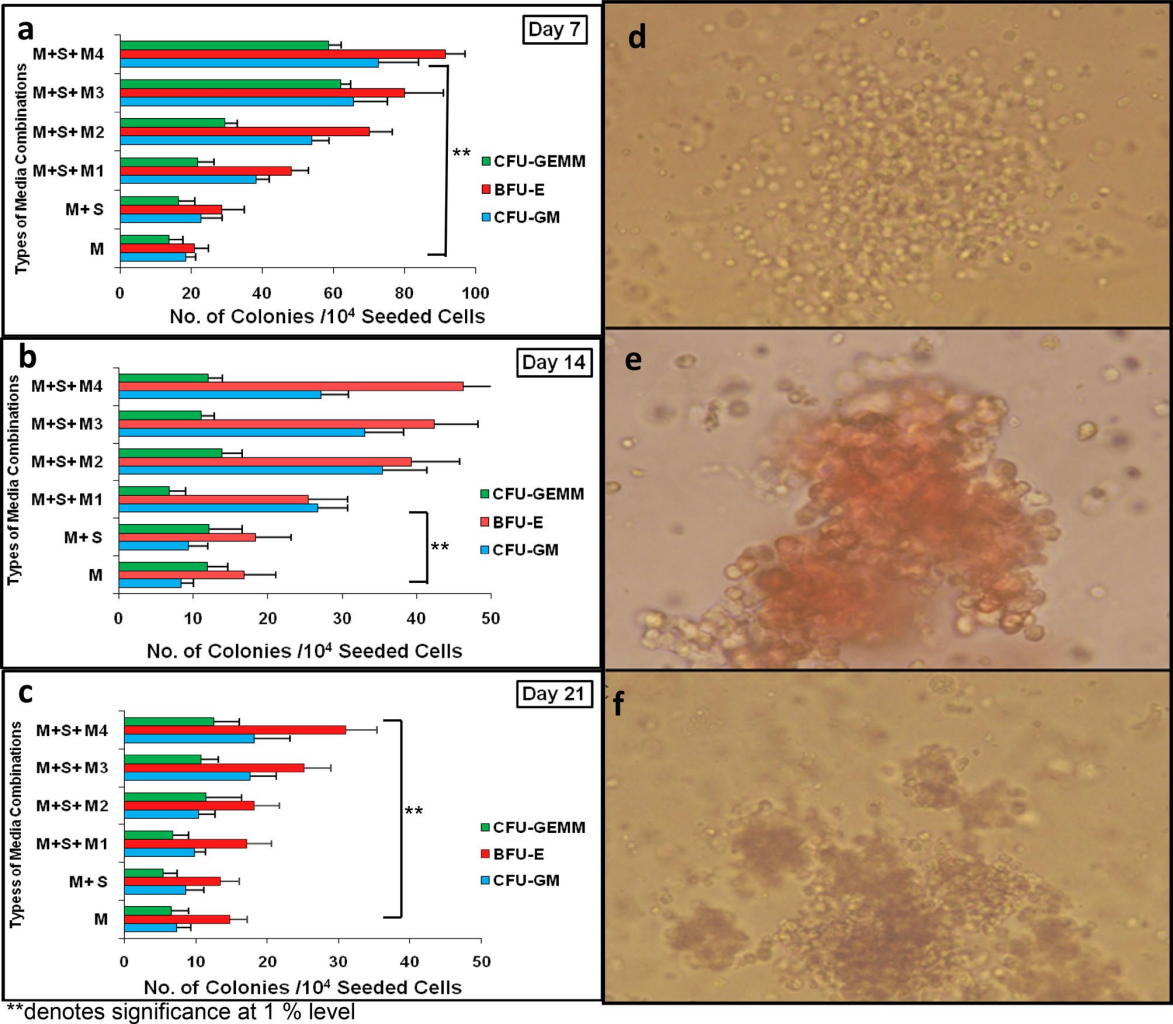
Colony Forming Unit Assay from *invitro* expanded FL CD34 + HSPCs . Number of committed progenitors (including CFU-GM, BFU-E, and CFU-Mix) derived from 1 × 10^4^ Fetal liver CD34^+^ HSPCs after 7 days (**a**), 14 days (**b**) and 21 days (**c**) of serum-free liquid suspension culture in the presence of different combinations of cytokines.M1, M2, M3 and M4 (n = 3). Statistical significance among colony forming potential from different media combinations as well as days was analyzed via ANOVA and is depicted with asterisks (*). double (**) asterisks indicate *P* ≤ 0.01. Representative Picture of CFU-GM (**d**), BFU-E (**e**) and CFU-Mix (**f**) colonies derived from Fetal liver CD34^+^ HSPCs (10×) from combination M + S + M4, day 14 of expansion.

**Figure 5:**
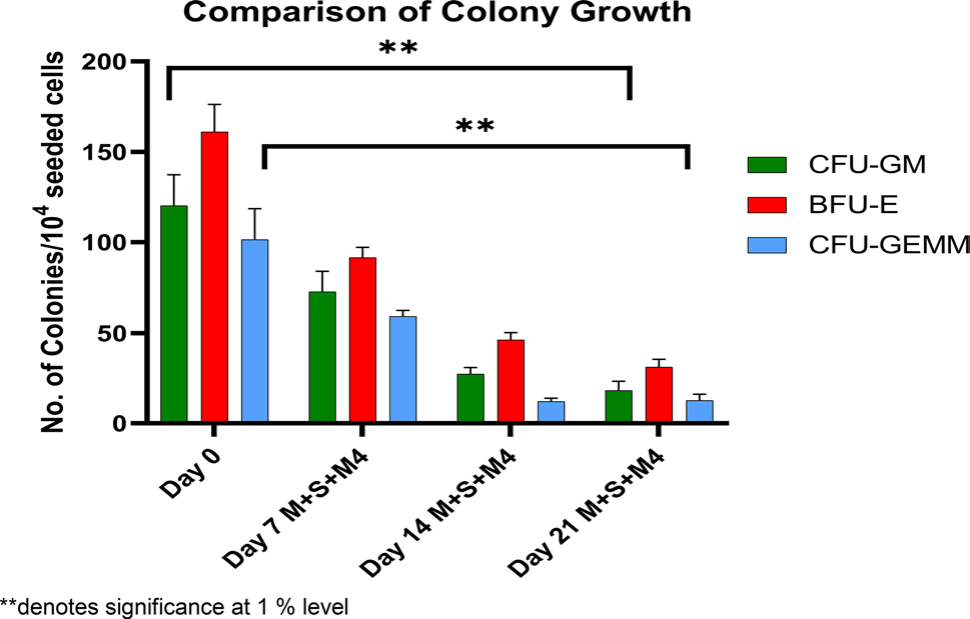
Colony Forming Unit Assay from M + S + M4 vs Day 0. Number of committed progenitors (including CFU-GM, BFU-E, and CFU-Mix) derived from 1 × 10^4^ freshly isolated Fetal liver CD34^+^ HSPCs, denoted as Day 0 vs. colonies generated from invitro expanded cells collected at day 7, day 14 and Day 21 from media combination M + S + M4 (n = 10). Statistical significance among colony forming potential from different media combinations as well as days was analyzed via ANOVA and is depicted with asterisks (*). double (**) asterisks indicate *P* ≤ 0.01.

In vitro expanded cells FL-HSPCs were counted and analyzed every 7 days for their colony formation (CFU-GM, BFU-E and CFU-GEMM) potential (Fig. 4a, b and c). The Colony forming potential of freshly isolated CD34^+^ HSPCs was 120 ± 17 for CFU-GM, BFU-E 161 ± 15 and 101 ± 17 for CFU-Mix. Figure 5 shows the comparison in different types of colonies generated from freshly isolated fetal liver CD34^+^ HSPCs (Day 0) with colonies generated from in vitro expanded cells collected at Day 7, 14 and Day 21 from media combination M + S + M4. Decline in colony forming potential was found to be significant (p-value (0.000) < 0.01) among different combination of media as well as day 14 and 21. Morphology of different colonies produced by expanded cells at Day 21 is shown in Fig. 4f.

## Discussion

HSPCs transplantation therapy has been effectively utilized for the treatment of several life-threatening hematopoietic diseases. Bone marrow (BM) and mobilized peripheral blood are the major sources of HSPCs; however, there is a paucity of human leucocyte antigen (HLA) matched donors. Hence, umbilical cord blood (UCB) has been used as an alternative to obtain HSPCs for transplantation therapy. UCB transplantation therapy has the limitation of availability of low numbers of HSPCs per unit of UCB. HSPCs from Fetal liver can be considered as potential candidates owing to their superiority in self renewal and differentiation potential. However, sample availability, as well as consistency of fetuses in terms of fetus age and HSPC count becomes a hurdle in using this important source. In vitro expansion of HSPCs from fetal liver has the potential to provide large number of cells useful for stem cell transplants as well as experimental studies. Possibility of in vitro expansion of HSPCs has been studied by several researchers to address this issue. Combination of various growth factors have commonly been employed to expand HSPCs in vitro. Mixture of stem cell factor (SCF), thrombopoietin (TPO), and FMS like tyrosine kinase 3 ligand (FLT-3L) was found to support the expansion of HSPCs. This study verified the effect of cytokines IL-6, FLT-3L, along with the role of serum supplementation in a short-term liquid culture^13^. Human recombinant FLT-3 ligand (50 ng/ml) in a stroma free long term cultures has been shown to be an early acting cytokine supporting the growth of very primitive HSPCs only when synergized with other growth factors^14^, including SCF (100 ng/ml)^15^, IL-3 (10 ng/ml)^16^, IL-6 (10 ng/ml)^17,18^, granulocyte colony stimulating factor (13 ng/ml) and 0.4 U EPO^19^ and 10U/ml TPO^14,20,21^. With these different combinations, expansion of CD34^+^ cells could be observed by day 7; expansion ranged from 2.44 to as high as 25.11 ± 13.50-fold by day 7^13,14,22,23^. Long term expansion of human fetal liver primitive hematopoietic progenitor cells in stroma free cultures was successfully achieved using media RPMI 1640 supplemented with 8% human AB plasma and a cocktail of different cytokines including FLT-3 ligand (50 ng/mL), interleukin 6 (10 ng/mL), megakaryocyte growth and development factor (MGDF; 10 ng/mL), and stem cell factor (SCF; 50 ng/mL)^24^. Combination of 5 cytokines namely SCF(100 ng/ml), FLT-3(100 ng/ml), IL-6(20 ng/ml), EPO (2U/ml) and GM-CSF(200U/ml) in a serum deprived culture medium STEM PRO-34-SFM supplemented with insulin, transferrin, low density lipoprotein, glutamine and mercaptoethanol has been used by us in the current study for a period of 21 days. Expansion of nucleated cells happened by 137.24 ± 8.1 fold (*p* < 0.05), levels of CD34^+^ antigen expression however, declined steadily over this period. It has been the general observation that even when there was clear demonstration of in vitro amplification of HSPCs, there was a net decline over input numbers (6.5 fold after 4 weeks) indicating that the culture conditions were suboptimal for stimulating HSPCs self-renewal and/or their continued survival (25). One possibility is that growth factors might positively alter self-renewal versus differentiation decisions of stem cells (25). Expansion of nucleated cells by 137.24 ± 8.1 fold in the present study but the loss of CD34^+^ cells by day 21 is certainly consistent with this possibility. Growth factor dosing is another important issue^25^. At high levels of human IL-3(20 ng/ml), GM-CSF (20 ng/ml), and SF (50 ng/ml), marked impairment of regeneration of human competitive repopulating cell (CRU) in NOD/SCID transgenic mice was noted by Nicolini et al.^26^. Concentrations and combination of different cytokines has proven their role in stem cell expansion studies. In one of the similar studies, although stimulation of HSPCs expansion by IL-11 was observed at low concentration range; yet saturation effects were not achieved even at high concentration with factors such as steel factor (SF) or FLT-3 ligand (FL), (> 300 ng/ml)^25^. According to McNiece et al*.,* SCF synergizes with other cytokines e.g., Epo, IL- 3, GM-CSF, and G-CSF to support growth of BFU-E, CFU-GM, and CFU-GEMM cells. SCF alone resulted in no significant colony formation, however, along with other cytokines viz. G-CSF and rhIL-3, SCF stimulated a synergistic increase in colony numbers^27^. A study by Sui et al*.,* reported that although SCF (100 ng/ml) alone has modest effect on colony growth, but in the presence of other cytokines like of lL-3 (200 U/ml), and EPO (2 U/ml), SCF increases both the size and the number of colonies^28^. Our study, similar to that of Matsunaga et al*.,* depicts that the combination of SCF (100 ng/ml)), IL-6 (100 ng/ml), and FLT-3 ligand (100 ng/ml) can support the proliferation, differentiation, and terminal maturation of BFU-E *‘*in vitro*’*, even in the absence of EPO^29^. Transforming growth factor beta 1 (TGF beta 1) at concentration of 1–50 pg/ml stimulated colony formation but at higher concentration, it had an inhibitory effect^30^. Stimulation of selective growth factor pathways, may yet be a crucial determinant of HSPCs self-renewal. For instance, regeneration of normal HSPCs in recipients lacking TPO got impaired by 10–20-fold^31^. TPO has been implicated as a positive regulator of HOXb4 (part of a family of transcription factors) expression, a potent enhancer of HSPCs expansion. HOXb4 induced self -renewal in vitro even in conditions that were sub optimal for untransduced HSPCs^25^. Over expression of another gene namely SALL4 has also been shown to have the capacity to substantially increase the number of HSPCs in vitro^32^. More recently, Jing et al.^33^ demonstrated the expansion of both murine BM HSPCs and human UCB HSCs with the combination of a moderate concentration of p38 inhibitor plus a GSK 3 inhibitor. Thus, it appears that despite the availability of various methods to expand HSPCs in vitro^25,32^, an ideal method to increase the number of HSPCs in vitro is yet to be discovered.

## Conclusion

Fetal liver, a major site of hematopoiesis during second trimester of pregnancy is an excellent source of hematopoietic stem cells. The higher cell number required for experimental studies is hindered by poor availability of fetuses and a smaller number of cells obtained per fetus. To overcome this problem, magnetically sorted CD34^+^ hematopoietic stem and progenitors cell populations have been subjected to expansion, using media supplements and different combinations of cytokines. Although the total number of nucleated cells increased in culture, levels of CD34 antigen expression declined steadily with time.

## Supplementary Information

Supplementary Information.

## Data Availability

All data generated or analyzed during this study are included in this “to be published” article.
