# Supplementary material for: In vitro expansion of fetal liver hematopoietic stem cells
Source: Sci Rep. 2021 Jun 4;11:11879. doi: 10.1038/s41598-021-91272-6 (PMC8178329; doi:10.1038/s41598-021-91272-6)
Supplement: Supplementary file 1 — Supplementary Information. [file 41598_2021_91272_MOESM1_ESM.pdf]

In vitro expansion of fetal liver hematopoietic stem cells

Authors :Rashmi Bhardwaj[1], Lalit Kumar[1], Deepika Chhabra[2]*, N K Mehra[1], Atul sharma[1], Sujata Mohanty[1] and Vinod Kochupillai[2]

Institutions [1]: Institute Rotary Cancer hospital (IRCH), All India Institute of Medical Sciences (AIIMS), New Delhi

[2]: Sri Sri Institute For Advanced Research (SSIAR), Ved Vignan Maha Vidhya Peeth (VVMVP), Bangalore

Corresponding author *: Deepika Chhabra. Ph.D. Microbiology, F003 Soudhamini Apartment, 21st Kanakpura Road, Art of Living International center, Udaipura, Bengaluru - 560082, +917696106221, chhabra.deepika@gmail.com

Supplementary Material

Table 1: Percentage of CD34$^+$ cells in FLMNCs at different gestational age groups

Gestational Age of the Fetus	Mean ± SD Value of CD34$^+$ cells %age	Range of CD34$^+$ cells %age
8 weeks	8.50±3.8%	5.2%-12.8%
9 weeks	8.70±1.8%	6.7%-10.2%
10 weeks	8.50±2.4%	6.8%-11.3%
11 weeks	6.83±1.2%	5.4%-7.9%
12 weeks	6.03±2.2%	3.6%-8.0%
13 weeks	4.40±1.3%	3.7%-5.5%
14 weeks	4.56±2.0%	2.3%-6.2%
15 weeks	2.73±0.9%	1.9%-3.8%
16 weeks	2.86±1.5%	1.2%-4.3%

Table 2: Total cell expansion and percentage of CD34+ cells in different cytokine mixtures at Day 21

**S. No.**	**M**		**M+S**		**M+S+M1**		**M+S+M2**		**M+S+M3**		**M+S+M4**	
	**Total cells×10^4**	**CD34+ cells (%)**	**Total cells×10^4**	**CD34+ cells**	**Total cells×10^4**	**CD34+ cells (%)**	**Total cells×10^4**	**CD34+ cells (%)**	**Total cells×10^4**	**CD34+ cells (%)**	**Total cells×10^4**	**CD34+ cells (%)**

				**(%)**								
**1**	**21.2**	**4.1**	**35**	**3.1**	**51.4**	**22.1**	**61**	**23.2**	**90.9**	**12.8**	**130**	**10.7**
**2**	**23.6**	**2.3**	**24.2**	**0.8**	**56.8**	**19.2**	**70.2**	**13.5**	**99.2**	**15.3**	**147**	**13.8**
**3**	**22.9**	**1.2**	**44.7**	**1.3**	**62.3**	**13.2**	**61.6**	**10.9**	**87.4**	**18.9**	**141.9**	**12**
**4**	**18.7**	**1.7**	**42.2**	**1.2**	**45.3**	**12.3**	**61.2**	**15.4**	**90.3**	**23.2**	**127.8**	**16.7**
**5**	**21.6**	**0.9**	**30.9**	**2.1**	**41.8**	**10.3**	**59.4**	**10.4**	**84.6**	**21**	**139.5**	**12.2**
**Median**	**21.6**	**1.7**	**35**	**1.3**	**51.4**	**13.2**	**61.2**	**13.5**	**90.3**	**18.9**	**139.5**	**12.2**
**Mean± SD**	**21.6 ± 1.8**	**1.7± 2.0**	**35.4 ± 8.4**	**1.7±1 .3**	**51.5±8. 3**	**15.4±4 .9**	**62.6± 4.2**	**14.6±5. 1**	**90.4± 5.4**	**18.2±4. 2**	**137.2 ±8.1**	**13.8±2. 3**
